# A history of late and very late stent thrombosis is not associated with increased activation of the contact system, a case control study

**DOI:** 10.1186/1477-9560-8-6

**Published:** 2010-04-15

**Authors:** Volker Pönitz, José W Govers-Riemslag, Hugo ten Cate, Rene van Oerle, Trygve Brügger-Andersen, Heidi Grundt, Patrycja Næsgaard, David Pritchard, Alf I Larsen, Dennis W Nilsen

**Affiliations:** 1 Institute of Medicine, University of Bergen, Bergen, Norway; 2 Department of Medicine, Stavanger University Hospital, Stavanger, Norway; 3 Laboratory for Clinical Thrombosis and Haemostasis, Department of Internal Medicine and Cardiovascular Research Institute Maastricht, Maastricht University, Maastricht, The Netherlands; 4 Axis-Shield, Dundee, UK

## Abstract

**Background:**

The pathophysiological pathways resulting in Late Stent Thrombosis (LST) remain uncertain. Findings from animal studies indicate a role of the intrinsic coagulation pathway in arterial thrombus formation, while clinical studies support an association with ischemic cardiovascular disease. It is currently unknown whether differences in the state of the contact system might contribute to the risk of LST or Very Late Stent Thrombosis (VLST). We assessed the relation between levels of several components involved in the contact system and a history of LST and VLST, termed (V)LST in a cohort of 20 patients as compared to a matched control group treated with PCI.

**Methods and Results:**

Activated factor XII (FXIIa), FXII zymogen (FXII), FXIIa-C1-esterase inhibitor (C1-inhibitor), Kallikrein-C1-inhibitor, FXIa-C1-inhibitor and FXIa-α1-antitrypsin (AT-inhibitor) complexes were measured by Enzyme-linked immunosorbent assy (ELISA) methodology.

Cases and controls showed similar distributions in sex, age, baseline medications and stent type. Patients with a history of (V)LST had a significantly greater stent burden and a higher number of previous myocardial infarctions than the control patients.

There were no significant between-group differences in the plasma levels of the components of the contact system.

**Conclusion:**

In a cohort of patients with a history of (V)LST, we did not observe differences in the activation state of the intrinsic coagulation system as compared to patients with a history of percutaneous coronary intervention without stent thrombosis.

## Introduction

Coronary stents are routinely employed in percutaneous coronary revascularization procedures and have significantly decreased the rates of acute vessel closure and restenosis[1]. The rate of stent thrombosis (ST) after percutaneous coronary intervention (PCI) is estimated to be as low as <1% of the cases following implantation of bare-metal stents (BMS), of which approximately 50% occur within the first month following implantation [2,3]. However, the potential fatality due to acute vessel closure, still makes ST one of the most frightening complications after percutaneous coronary intervention (PCI) [4,5]. During the last years, concerns have been raised regarding later occurrence of ST in drug-eluting stents (DES), in particular beyond the traditional 1-month timeframe[6-8]. The actual incremental risk associated with DES has been an issue of controversy.

Among others, discontinuation of antiplatelet therapy, increased intrinsic platelet activity, incomplete stent apposition, bifurcation stenting, renal insufficiency and diabetes have been discussed as possible risk factors for the development of late stent thrombosis (LST) (1-12 months) and very late stent thrombosis (VLST) (>12 months), and local inflammatory processes have been shown to be associated with (V)LST in autopsy materials [9-12]. However, the occurrence of (V)LST most likely has a multifactorial origin and the exact pathophysiological scenario leading to (V)LST remains uncertain.

Currently there is no established method to identify patients at high risk of (V)LST.

Contact activation, believed to be potentiated by negatively charged surfaces, results in the activation of the zymogen FXII to FXIIa and affects several pathophysiological processes including hypotension, inflammation, thrombosis and fibrinolysis[13-20].

Several components of the contact system have been found to be related to coronary heart disease (CHD) [21-27] and recent findings from animal studies indicate that FXII might even be essential for arterial thrombus formation[28].

However, in contrast, other clinical studies have demonstrated low FXII levels as a risk factor for coronary heart disease or showed a U-shaped association to risk[29-31]. Clinical settings and patient populations differ substantially between the existing clinical studies, and even if an association of the contact system to CHD and thrombosis seems likely, the exact mechanisms remain poorly understood. Whether the activation state of the contact system might be associated with the occurrence of (V)LST has never been addressed.

The objective of the present study was to test the hypothesis that patients having survived an event of (V)LST have a generally different activation state of the contact system as compared to matched patients without a history of this condition.

## Methods

### Study design and patient population

The study was a single-centre cohort study (Risk markers for Late in Stent Thrombosis (RIST)), in which 20 patients with a history of definitive (V)LST (as proposed by the Academic Research Consortium [32]) were compared to 32 PCI controls without (V)LST. These individuals were matched with respect to age, sex, current antiplatelet medication and stent type [Seventy-five percent of the (V)LST patients and 84.4% of the controls treated with DES (TAXUS EXPRESS). The remaining patients received BMS (Flexmaster or Express stents)]. Patients were included at the Stavanger University Hospital, Norway in March 2008.

All patients who could be identified from a local PCI registry with a history of a definite (V)LST admitted during the last 4 years were included in the study. Exclusion criteria were age below 18 years or unwillingness or inability to provide informed consent.

Assessment of previous clinical history, current and previous medication and risk factors were based on hospital records, angiographic records and personal interview. Additionally, hospital journals were searched for confirmation of reported data. Diabetes mellitus was defined as either whole blood fasting glucose concentrations above 6.1 mmol/L, two hour post glucose load concentrations above 10.0 mmol/L or medically treated diabetes mellitus, and hypercholesterolemia was defined as total cholesterol concentrations above 6.5 mmol/L or medically treated hypercholesterolemia. Arterial hypertension was defined as repeated blood pressure measurements above 140/90 mmHg or treated hypertension.

Written informed consent was obtained from all patients. The study was approved by the Regional Board of Research Ethics and the Norwegian Health authorities and conducted in accordance with the Helsinki declaration of 1971, as revised in 1983.

### Blood Sampling Procedures and Laboratory Measurements

Blood samples were drawn by puncture of an antecubital vein with minimal stasis and centrifuged twice for 15 minutes at 2.000 × g and for 10 minutes at 11.000 × g at 18°C. Laboratory measurements were either performed immediately following centrifugation or following storage of the samples at -80°C.

A measure of FXIIa in citrated plasma was obtained by enzyme linked immunosorbent assay (ELISA methodology), using a specific monoclonal capture antibody (Mab 2/215) with no detectable recognition of FXII or FXIIa-C1 inhibitor complexes [33]. The alkaline phosphatase conjugated secondary antibody was a sheep polyclonal antibody against FXIIa. Kits were supplied by Axis-Shield, Dundee, United Kingdom.

FXII was measured in citrated plasma using ELISA, based on a sheep polyclonal anti-FXII capture antibody and a horseradish peroxidase conjugated goat polyclonal anti-FXII secondary antibody (*Enzyme Research Laboratories*).

Complexes of FXIa, FXIIa, Kallikrein and C1-inhibitor and complexes of FXIa and AT-inhibitor were measured in EDTA plasma, to which soybean trypsin and benzamidin (*Sigma-Aldrich*) was added to prevent contact activation during collection and processing, with enzyme-linked immunosorbent assays (ELISAs), as previously described with minor modifications[27]. The detection limits were 0.095% for all four assays, respectively.

Hs-CRP was quantified in serum by a Tina quant^® ^C-reactive protein (latex) high sensitivity assay (*Roche Diagnostics*) [34].

### Statistical methods

Approximately normally distributed variables were given as mean and standard deviation, whilst skewed distributions were given as median and interquartile range. For measurements of inhibitor complexes, values below the cut-off limit of 0.095% were set to 0.095. The two sample t-test was used to examine differences between the patient and control group for normally distributed variables, whilst the Mann-Whitney-U test was applied to test for the equality of the median of two samples of variables with a skewed distribution. The Chi-square test for association was applied for baseline categorical variables. The statistical analyses were performed using SPSS version 15.0. In all tests a two-tailed P-value of < 0.05 was considered to statistically significant.

## Results

21 patients with a history of (V)LST were identified from our PCI registry. One patient was not included in the study due to unwillingness to participate. The total study population consisted of 52 patients. Twenty patients suffered from a definite late or very late stent thrombosis within the last 43 (median 10) months and there were 32 PCI controls without (V)LST (median follow-up time 15 months). Clopidogrel had been discontinued in all patients prior to the development of (V)LST and after the recommended treatment time of 6 - 12 months, with a median discontinuation period of 202 days, whereas all were receiving acetylsalicylic acid (ASA). Ninety percent of the (V)LST patients were back on dual antiplatelet therapy consisting of ASA and clopidogrel when they were tested, and 91% of controls had been on continuous dual antiplatelet treatment. The remaining 10 and 9%, were only treated with ASA at the time of testing.

Baseline variables and demographics for the two groups are displayed in Table 1 and baseline biochemical variables are displayed in Table 2. There was no significant difference in the incidence of multivessel disease (50% vs 40.6%; p = 0.57) or minimum stent diameter 2.8 ± 0.4 mm vs 2.9 ± 0.4 mm (Mean ± SD; p = 0.28) between the (V)LST and the PCI control group. The total stent burden was significantly greater in the (V)LST group as compared to the PCI control group (total stent length 36.0 (30.5-48.0 mm) vs 20.0 (16.5-28.0 mm) (Median (25-75% percentiles; p < 0.01). Specific variables related to (V)LST are displayed in Table 3.

**Table 1:** Baseline clinical characteristics for patients with a history of late or very late stent thrombosis (LST/VLST) and controls.

Characteristics	(V)LST group (n = 20)	PCI Control group (n = 32)	P
*Demographics*			
Age (years)*	53.8 ± 8.6	56.9 ± 9.7	0.25
Female gender, n (%)	4 (20)	8 (25)	0.75
BMI kg/m^2 ^*	26.0 ± 3.6	25.9 ± 3.7	0.87
Alcohol consumption (U/week)*	3.8 ± 3.7	3.1 ± 3.4	0.50
Current smoking n (%)	7 (35)	9 (28)	0.76
Previous smoker n (%)	11 (55)	19 (59)	0.78
Angina pectoris (%)	3 (15)	7 (22)	0.72
Congestive heart failure n (%)	2 (10)	2 (6)	0.63
Hypertension n (%)	5 (25)	12 (38)	0.38
Diabetes mellitus n (%)	3 (15)	5 (16)	1.00
Hypercholesterolemia n (%)	9 (45)	11 (34)	0.56
Coagulation disorders n (%)	1 (5)	0 (0)	0.39
Current active infections n (%)	2 (10)	4 (13)	1.00
Chronic inflammatory disorders n (%)	1 (5)	5 (16)	0.39
Antiphospholipid syndrome n (%)	0	0	1.00
Pregnancy n (%)	0	0	1.00
Active malign disease n (%)	0	0	1.00
Previous MI n (%)	20 (100)	31 (97)	1.00
No of previous MI (n)*	1.8 ± 0.6	1.1 ± 0.4	<0.01
Family history of CHD n (%)	15 (75)	21 (66)	0.55
Previous VTE n (%)	0	0	1.00
Family history of VTE n (%)	5 (25)	5 (16)	0.48
Previous stroke n (%)	1 (5)	0 (0)	0.39
Previous PAD n (%)	1 (5)	1 (3)	1.00
Coronary artery bypass graft n (%)	1 (5)	2 (6)	0.85
*Current medication*			
Acetylsalicylic acid n (%)	20 (100)	32(100)	1.00
Clopidogrel, n (%)	18 (90)	29 (91)	1.00
Warfarin n (%)	0	0	1.00
Angiotensin converting enzyme inhibitor n (%)	11(55)	15(47)	0.78
Angiotensin-2 receptor blocker, n (%)	5 (25)	10 (31)	0.76
Beta blocker n (%)	9 (45)	12 (38)	0.77
Calcium channel blocker n (%)	0 (0)	2 (6)	0.52
Aldosteron antagonist n (%)	5 (25)	3 (9)	0.24
Insulin n (%)	1 (5)	0 (0)	0.39
Oral antidiabetics n (%)	1 (5)	4 (13)	0.64
NSAID n (%)	0 (0)	1 (3)	1.00
Statin n (%)	19 (95)	31 (97)	1.00
Hormone replacement therapy n (%)	0 (0)	1 (3)	1.00

*Mean ± SD; definer her diabetes, hyperkolesterolemi, family history of MI etc fra RACS)

**Table 2:** Baseline biochemical characteristics for patients with a history of late or very late stent thrombosis (LST/VLST) and controls.

Characteristics	(V)LST group (n = 20)	PCI Control group (n = 32)	P
FXIIa (pM)*	70.0 (52.0-91.0)	67.5 (54.0-86.3)	0.95
FXII (%)*	99.0 (95.3-103.8)	99.0 (93.5-102.8)	0.79
FXIa-C1-inhibitor complex (%)*	0.17 (0.11-0.26)	0.21 (0.13-0.43)	0.25
FXIa-AT -complex (%)*	0.19 (0.15-0.27)	0.20 (0.15-0.28)	0.49
FXIIa-C1-inhibitor complex (%)*	0.11 (0.10-0.25)	0.13 (0.10-0.24)	0.80
Kallikrein-C1-inhibitor complex (%)*	0.10 (0.10-0.12)	0.10 (0.10-0.11)	0.93
Hs-CRP mg/L *	1.3 (1.0-3.1)	2.15 (1.0-3.8)	0.41
Se-creatinine (μmol/L)†	83.1 ± 18.0	85.9 ± 21.2	0.62
eGFR (ml/min)†	83 ± 19	79 ± 19	0.40
Hemoglobin g/dl†	14.0 ± 1.5	14.0 ± 1.4	0.94
Hematocrit %†	42.2 ± 4.1	41.8 ± 3.8	0.75
Thrombocytes (10^9^/L)†	251 ± 42	257 ± 79	0.78
Leucocytes (10^9^/L)†	6.6 ± 1.5	7.2 ± 2.4	0.25
Neutrophil count (10^9^/L)†	3.8 ± 1.0	4.4 ± 1.8	0.21
Basophil count (10^9^/L)†	0.01 ± 0.04	0.01 ± 0.03	0.95
Eosinophil count (10^9^/L)†	0.23 ± 0.21	0.21 ± 0.12	0.61
Total Cholesterol (mmol/L)†	3.80 ± 0.61	3.96 ± 0.92	0.66
LDL-Cholesterol (mmol/L)†	1.8 ± 0.6	1.8 ± 0.6	0.91
HDL-Cholesterol (mmol/L)†	1.33 ± 0.28	1.36 ± 0.75	0.91
Triglycerides (mmol/L)†	1.4 ± 0.8	1.7 ± 0.8	0.27
HbA1C % †	5.7 ± 0.8	5.8 ± 0.7	0.88

* Median (25-75% percentiles); † Mean ± SD

**Table 3:** Characteristics of patients with (V)LST.

**Characteristic**	
	
Discontinuation of clopidogrel, n (%)	20 (100)
Clopidogrel free interval prior to (V)LST (days, median, range)	202 (1433)
Discontinuation of acetylsalicylic acid n (%)	2 (10)
Acetylsalicylic acid free interval prior to (V)LST (days, median, range)	0 (180)
Late Stent thrombosis (6-12 months) n (%)	6 (30)
Very late stent thrombosis (> 12 months) n (%)	14 (70)
*Vessel with stent thrombosis*	
*LAD n (%)*	12 (60)
*CX n (%)*	3 (15)
*RCA n (%)*	5 (25)
	
Multivessel stenting prior to LST n (%)	3 (15)

However, for none of the investigated components of the contact system, significant differences could be observed between the groups (Table 2).

## Discussion

In the present study we could not demonstrate differences in the activation state of the contact system after measuring several different components of the intrinsic coagulation and the plasma kallikrein-kinin system (PKKS) in patients with a history of (V)LST as compared to controls.

Stent thrombosis is currently considered a multifactorial problem related to patient, lesion, procedure, factors related to blood coagulation and response to antiplatelet therapy[3]. For understandable reasons much attention has been on the contribution of platelets and the (lack of) efficacy of platelet inhibiting agents. Stent thrombosis, in particular following the implantation of DES, has been mainly attributed to discontinuation or premature cessation of clopidogrel[5,35]. However, dual antiplatelet therapy has not been found to entirely prevent the occurrence of LST[36]. In addition, the occurrence of ST does not seem to be strictly related to the time point of clopidogrel cessation, as the event often occurs a long time after its cessation. Moreover, the majority of patients do not develop ST despite premature cessation of antiplatelet medication. Consequently, it seems obvious that other factors besides discontinuation of antiplatelet therapy may be involved in the pathogenesis of ST.

Renne et al. provided a challenging new concept for FXII in arterial thrombosis, showing impaired thrombus stability in FXII deficient mice[28]. In addition they showed that FXII deficient mice were partly protected against ischemic stroke. Moreover, numerous clinical studies have reported a role of different components of the contact system and the PKKS in CHD, but detailed pathophysiological mechanisms still remain poorly understood. Increased FXII zymogen levels have primarily been associated with a decrease in risk for MI and outcome [29,31]. In contrast, elevated plasma FXIIa has been shown to be associated with an increased risk of CHD, the extent of coronary atheroma and with survival status following an MI compared to healthy controls[21-24]. Moreover, FXIIa measured after an MI independently predicted recurrent coronary occlusive events, [25] and FXIIa in chest pain patients at hospital admission predicted long-term mortality [26] whereas other studies failed to show clear associations of FXIIa to CHD[30]. Measurement of inhibited FXIIa (as the FXIIa-C1-inhibitor complex) suggested that the level of this inhibitor complex was associated with cardiovascular risk [27].

To our knowledge, late- and very late stent thrombosis have not been described in relation to the contact and/or plasma kallikrein-kinin system yet. In our analyses, we examined different components of these systems and their possible association to (V)LST. Low levels of inhibitor complexes for the PKKS in both groups reflect a high general risk for CHD [27]. However, for none of the measured biochemical variables, significant differences between patients with a history of (V)LST and controls were noted. Consequently, our results do not suggest a role of the contact system in the pathogenesis of (V)LST.

The design of our study carries along several issues that have to be considered with caution. Our ST population consisted of patients suffering from both late- and very late stent thrombosis, as well as of patients with implantation of both DES and BMS. The pathophysiology of ST may differ between these settings and may also lead to a different involvement of the contact system.

The main limitation of the study is its retrospective design, with no knowledge of the state of the contact system at the time of the event. Ideally, also the discontinuation period for antiplatelet medication should have been matched between the case and control group. However, as most patients with a history of LST receive double antiplatelet medication for an indefinite time period in contrast to cessation of clopidogrel nine months to two years following uncomplicated stent implantation, an optimal matching regarding this parameter could not be achieved.

In addition, the results of the present study are limited by the restricted sample size. To achieve a statistical power of 80% at a significance level of 5%, large differences in variables between the groups would have been necessary, for example 7% for FXII, 31% for FXIIa, and 53% for FXIIa-Kallikrein-inhibitor. Whether potentially smaller changes may be of biological importance is not known and cannot be excluded by our data.

## Conclusion

The present study does not suggest an association of the activation state of the intrinsic coagulation system in relation to the occurrence of (V)LST.

## Funding

This work was supported by a grant of the Stavanger University Hospital Research Foundation.

## Conflicts of interests

Co-author David Pritchard is employee at AXIS-Shield, UK.

## Authors' contributions

VP participated in the design and conduction of the study and data collection, performed the statistical analysis and is main author of the manuscript. JG carried out the ELISAs on inhibitor complexes of the PKKS and helped to draft the manuscript. HtC participated in the design of the study and helped to draft the manuscript. RvO participated in the conduction of the study and data collection. TBA participated in the conduction of the study, data collection and helped to draft the manuscript. HG participated in the design of the study and assisted in the performance of the statistical analyses. PN participated in the conduction of the study and data collection. DP carried out the ELISAs on FXII and FXIIa and helped to draft the manuscript. AIL provided intellectual contributions in the interpretation of the angiographic data and helped to draft the manuscript. DN participated substantially in the design of the study and helped to draft the manuscript. All authors read and approved the final manuscript.
